# A commentary on ‘Meta-research studies in surgery: a field that should be encouraged to assess and improve the quality of surgical evidence’

**DOI:** 10.1097/JS9.0000000000000800

**Published:** 2023-10-04

**Authors:** Si-Un Frank Chiu, Ru-Yi Huang, Chong-Chi Chiu

**Affiliations:** a Department of Computer Science; b Department of Economics, Brown University, Providence, Rhode Island, USA; c Department of Family Medicine; d Department of Medical Education and Research; e Department of General Surgery, E-Da Cancer Hospital; f School of Medicine, College of Medicine, I-Shou University, Kaohsiung, Taiwan

*Dear Editor,*

The continuous growth of new knowledge is pushing the boundaries of surgical disciplines, and numerous review articles try to consolidate this vast expansion of information. However, compared to other medical fields, surgical specialties have historically needed to be faster in embracing evidence-based medicine, primarily because of the exclusive challenges while conducting surgical clinical trials. These challenges also spread out to systematic reviews and meta-analyses, where arguments on the quality and heterogeneity of included articles pose significant concerns. Thus, the recent perspectives shared by Narvaez-Rojas *et al*.^1^ should raise a red flag among surgeons.

Meta-analyses prioritize the characteristics of seeking, summarizing, and interpreting medical literature on a specific topic using rigorous statistical approaches. Dr Glass (Gene V. Glass) developed this quantitative research synthesis technique in 1975, and it has now become an essential part of the research armamentarium. Nevertheless, conducting systematic reviews and meta-analyses of surgical interventions comes with unique challenges and considerations, which would impact the validity and applicability of the results^2^. For example, studies with heterogeneous populations can result in unexplained heterogeneity in meta-analyses, further raising doubts about the reliability of the reviews. Surgical interventions encompass multiple facets of patient care, including preoperative assessment, surgical technique, anesthesia, and postoperative care, all of which can influence outcomes and introduce heterogeneity. Additionally, surgeon preferences and learning curves can affect the comparability of different surgical interventions, while patient preferences may present challenges in recruiting participants for surgical trials. Furthermore, blinding participants and investigators in surgical interventions is particularly challenging, leading to potential bias, and conducting trials in emergency settings raises ethical and recruitment difficulties. Lastly, industry involvement in funding surgical trials may be associated with outcome bias. To effectively address heterogeneity, it is imperative to select appropriate statistical models while meticulously considering the study-level factors.

The compromised validity of these systematic reviews and meta-research publications restricts their usefulness for clinical, educational, research, and medical policy purposes. Therefore, conducting well-designed systematic reviews is of utmost importance for evidence-based practice in surgery, enabling the identification of evidence gaps and guiding future research directions. Although there has been an improvement in the quality of clinical trials and systematic reviews in surgery, there remains room for further progress. Recently, Crocker *et al*.^3^ advocated the application of version 2 of the Cochrane risk-of-bias tool (RoB 2) to ensure and enhance the quality of systematic reviews and meta-analyses. Some experts believe it could improve the validity assessment to evaluate the quality of the collected articles. Furthermore, lessening the ascertainment and selection bias could be achieved through adjudication, blinding of assessors, using various assessors, and comprehensive evaluation. Besides, transparent demonstration of search databases such as Web of Science, ScienceDirect, Scopus, Medline (PubMed), Embase, ProQuest, and other bibliographies, searching unprinted articles, contacting the corresponding authors, handling artificial intelligence-assisted searching, and incorporating various language studies, should help reduce bias. Methodological instructions, like those manifested in the Quality of Reporting of Meta-analyses (QUOROM) comment, are pivotal in mitigating bias and improving the reporting of meta-analyses.

Sauerland and Seiler^4^ highlighted the crucial position of systematic reviews and meta-analyses in evidence-based research for surgeons, offering insights into designing and conducting such research. Systematic reviews and meta-analysis studies are at the pinnacle of the pyramid, representing the most substantial scientific evidence and providing highly efficient reference material for establishing health policy and clinical practice. They offer precise evidence about disease status, incidence, prevalence, correlations, and other critical aspects, often surpassing the information gleaned from clinical trial studies. Fortunately, many burdens while performing a systematic review are docile to automation empowered by artificial intelligence^5^, such as reviewing the article content, for example, title, abstract, entire content of studies included, extracting data, and collating the results of a meta-analysis. Leveraging the power of text mining, natural language processing, and the advancement of machine learning has led to a pioneering method that could accurately imitate human efforts in performing a systematic review but at a faster and more cost-effective pace.

Overall, well-conducted systematic reviews in surgery provide invaluable guidance for clinical practice. However, to ensure their effectiveness, authors must adhere to rigorous systematic review methods, peer reviewers should give constructive criticism, and editors must pay attention to the recommendations outlined in the QUOROM statement. Moreover, surgeons must understand the strengths and weaknesses of this method to critically assess the findings, as the results of a meta-analysis can significantly impact clinical practice. Continued improvements in the quality of surgical clinical trials and the assistance of artificial intelligence in systematic reviews are expected to advance evidence-based surgical interventions.

## Ethical approval

This is only a commentary, not research involving patients. No ethical approval is required.

## Consent

This is only a commentary, not research involving patients. No patient consent is required.

## Sources of funding

This is a commentary. We have no funding for our commentary.

## Author contribution

S.-U.F.C.: conceptualization and writing the original draft; R.-Y.H.: validation; C.-C.C.: conceptualization, supervision, submission, and correspondence.

## Conflicts of interest disclosure

There is no conflict of interest in our commentary.

## Research registration unique identifying number (UIN)

This is a commentary on a study published in ‘International Journal of Surgery’. No UIN is required.

## Guarantor

Professor Chong-Chi Chiu.

## Data availability statement

This is only a commentary on a study published in ‘International Journal of Surgery’. There is no research data in our commentary.

## Provenance and peer review

This paper was not invited.
